# More than one way to see it: Individual heuristics in avian visual computation

**DOI:** 10.1016/j.cognition.2015.05.021

**Published:** 2015-10

**Authors:** Andrea Ravignani, Gesche Westphal-Fitch, Ulrike Aust, Martin M. Schlumpp, W. Tecumseh Fitch

**Affiliations:** a Department of Cognitive Biology, Faculty of Life Sciences, University of Vienna, Althanstrasse 14, 1090 Vienna, Austria; b Language Evolution and Computation Research Unit, University of Edinburgh, EH8 9AD Edinburgh, UK; c Haidlhof Research Station, University of Vienna/University of Veterinary Medicine Vienna/Messerli Research Institute, 2540 Bad Vöslau, Austria

**Keywords:** Artificial grammar learning, Local/global processing, Species differences, Model selection, Maximum likelihood, Language evolution

## Abstract

Comparative pattern learning experiments investigate how different species find regularities in sensory input, providing insights into cognitive processing in humans and other animals. Past research has focused either on one species’ ability to process pattern classes or different species’ performance in recognizing the same pattern, with little attention to individual and species-specific heuristics and decision strategies. We trained and tested two bird species, pigeons (*Columba livia*) and kea (*Nestor notabilis*, a parrot species), on visual patterns using touch-screen technology. Patterns were composed of several abstract elements and had varying degrees of structural complexity. We developed a model selection paradigm, based on regular expressions, that allowed us to reconstruct the specific decision strategies and cognitive heuristics adopted by a given individual in our task. Individual birds showed considerable differences in the number, type and heterogeneity of heuristic strategies adopted. Birds’ choices also exhibited consistent species-level differences. Kea adopted effective heuristic strategies, based on matching learned bigrams to stimulus edges. Individual pigeons, in contrast, adopted an idiosyncratic mix of strategies that included local transition probabilities and global string similarity. Although performance was above chance and quite high for kea, no individual of either species provided clear evidence of learning exactly the rule used to generate the training stimuli. Our results show that similar behavioral outcomes can be achieved using dramatically different strategies and highlight the dangers of combining multiple individuals in a group analysis. These findings, and our general approach, have implications for the design of future pattern learning experiments, and the interpretation of comparative cognition research more generally.

## Highlights

• Two bird species are trained and tested on a visual sequence learning task.
• We infer individual decision heuristics using a model selection approach.
• Birds show differences in number, type and heterogeneity of decision strategies.
• Parrots use few, focused decision rules; pigeons an idiosyncratic mix of strategies.
• No bird convincingly masters the target pattern, although all perform above chance.

## Graphical abstract

**Figure:**
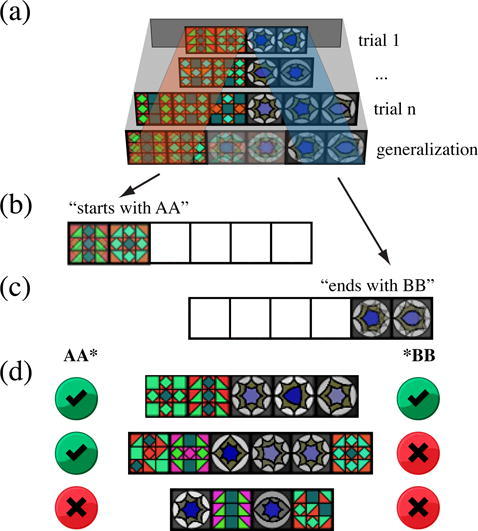

## 1 Introduction

### 1.1 Processing of sensory regularities by humans and other animals

Humans are strongly inclined to discover and process structure in sensory stimuli (Gombrich, 1984). Appreciating the overall symmetry of a building or painting, delighting in themes and variations in music, or parsing a sentence in our native language are all examples of tasks that require sophisticated structural processing. Whether natural or man-made, complex visual, auditory or tactile inputs are usually categorized by humans using relations established between their constituent components (Conway & Christiansen, 2005). Such structure-based learning is an important part of humans’ everyday sensory experience, regardless of whether these learning processes are statistical or explicitly rule-based (Peña, Bonatti, Nespor, & Mehler, 2002).

Some cognitive resources required to process structure are shared across domains (e.g., music and language) and possibly with other animal species. Aspects of human working memory capacities, for example, appear to be both domain-general (Chiappe and MacDonald, 2005, Janata et al., 2002, Kirkham et al., 2002) and shared with a broad range of animal species (Chiappe and MacDonald, 2005, Kawai and Matsuzawa, 2001, Murphy et al., 2008). Similarly, several species can learn that some pairs of events co-occur more often than others (see ten Cate & Okanoya, 2012 for an overview of how other animals process transitional probabilities). However, some structural computations at the core of human cognition may be difficult or even impossible for other animals to process. Empirical investigations of human specificity require use of the comparative method (Fitch, 2014, Fitch et al., 2010), where different species are tested on matched tasks to draw biological inferences concerning a particular cognitive trait. This method can, for example, be applied to estimate which cognitive prerequisites for language or music emerged during recent human evolution and which arose earlier in primate, mammal or vertebrate evolutionary history (Fitch, 2005).

### 1.2 Processing “context-free” structures across species and domains

Pattern perception experiments can be formalized using quantitative frameworks. For example, formal language theory is a branch of mathematics and computer science that offers analytical tools to measure complexity of structural patterns (strings composed of minimal holistic elements (Jäger & Rogers, 2012). Formal language theory has recently been adopted in perceptual experiments in humans and non-human animals (Fitch and Friederici, 2012, ten Cate and Okanoya, 2012). Formal language theory provides a rigorous mathematical framework and non-ambiguous notation to clearly state hypotheses and to sharpen research questions (Fitch, 2014). Researchers choose some abstract rule system or “grammar” of interest, and use it to produce visual or auditory test stimuli that either follow or violate the rule(s) (Jäger and Rogers, 2012, Reber, 1969).

**Fig. 1:**
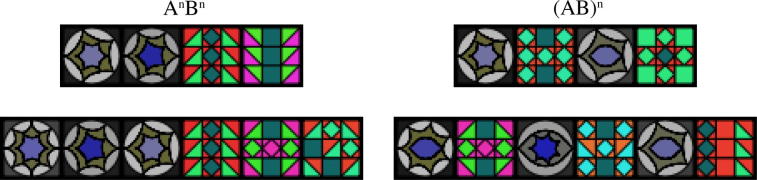
Example of training patterns. Top: an AABB (equivalent to A^2^B^2^, left) and ABAB (equivalent to (AB)^2^, right) pattern; bottom: same patterns for *n* = 3. During the training, birds were simultaneously presented with an A*^n^*B*^n^* and an (AB)*^n^* stimulus (with *n* = 2 or 3) and were rewarded for pecking on one of them, depending on the experimental group to which they were randomly assigned.

One controversial strand of comparative pattern learning research concerns animals’ ability to process supra-regular (e.g., “context-free”) structures, which incorporate relationships between multiple non-adjacent elements. Mastery of such relationships is a necessary (but not sufficient) prerequisite for using human language (Fitch & Friederici, 2012). An early study compared pattern-learning abilities in humans and cotton-top tamarins (Fitch & Hauser, 2004) using syllable streams that either followed an *alternating* pattern (female–male–female–male, etc., notated as (AB)*^n^*), or a *matched block* pattern (female–female–…–male–male…, notated A*^n^*B*^n^*). In terms of formal language theory, processing the alternating stimulus requires weaker computational capabilities than the block pattern (see Fig. 1 for a visual equivalent of these two types of pattern). While humans could readily discriminate both syllable patterns, the monkeys only mastered the less computationally demanding alternating pattern (Fitch & Hauser, 2004). A further study, using operant testing, investigated whether starlings could learn the same block pattern (A*^n^*B*^n^*) composed of starling vocalizations (Gentner, Fenn, Margoliash, & Nusbaum, 2006). After intensive training, the birds discriminated such patterns from ill-formed variations, featuring different combinations and orderings of the constituent starling calls (Gentner et al., 2006). The apparently superior performance of starlings over monkeys could represent a species difference reflecting the complexity of starlings’ natural vocalization (although for counter-hypotheses see ten Cate and Okanoya, 2012, van Heijningen et al., 2009). However, starlings underwent an extensive training period, unlike the monkeys, who received no feedback or training.

Similar patterning abilities were subsequently investigated in zebra finches, a bird species that exhibits a relatively simple song structure (van Heijningen et al., 2009). Although a group-level data analysis suggested that, like starlings, zebra finches mastered the intended “complex” pattern, a more detailed analysis of the performance of individual birds revealed that each bird was using a simple rule, often only taking a tiny portion of the chosen stimulus into account. van Heijningen et al. (2009) thus concluded that none of their zebra finches actually learned the grammar, despite apparent success at a group level, and suggested that the same reasoning could be applied to the previous starling results (Gentner et al., 2006). The dispute has not yet been resolved and has given rise to debate (Gentner et al., 2010, ten Cate et al., 2010) and additional studies in further species (Abe and Watanabe, 2011, Rey et al., 2012, Stobbe et al., 2012).

Thus, although a number of species can parse “computationally simple” stimuli, corresponding to regular languages at the lowest level of the formal language hierarchy (Abe and Watanabe, 2011, Fitch and Hauser, 2004, Gentner et al., 2006, Herbranson and Shimp, 2008, Ravignani et al., 2013, ten Cate and Okanoya, 2012), perception of patterns at higher complexity levels – supra-regular (Jäger & Rogers, 2012), i.e., beyond simple chaining of contiguous perceptual tokens – has yet to be convincingly demonstrated in non-human animals. Thus, there is still no consensus on (a) which nonhuman species, if any, can master supra-regular rules and (b) precisely how complex patterning rules are learned and processed in cognitive experiments. The latter question is the focus of the research described here.

### 1.3 Artificial grammar learning: Common problems need a novel solution

Previous research has shown that a number of factors can influence the overall outcome of pattern learning experiments. Participants may achieve (partial) success in these experiments by using simple heuristics and perceptual shortcuts, rather than learning the intended abstract rules (van Heijningen et al., 2009). During training, participants may learn some simple “heuristic” or “strategy”, which although simpler than the “correct” rule, nonetheless earns them rewards and results in above-chance performance. When this heuristic is applied to novel test stimuli where it does not fit, misclassification results. Detailed analysis of individual participants’ responses to patterns of similar length, but inconsistent with the generating rule, are necessary to determine which particular decision strategy was used by a participant during the training phase. This type of analysis is particularly suitable for the large quantities of data resulting from operant conditioning experiments like the ones described here.

When trained and tested on pattern perception experiments, two species may perform differently because only one learned the abstract rule, or found an efficient behavioral shortcut. Alternatively, two species might show similar overall performances, although with one mastering an abstract rule and the other using a simpler but efficient shortcut. Individual differences in strategy may be crucial in this, because merged group data often describe a fictitious “average learner” that corresponds to no individual participant (Zimmerer et al., 2011, Zuidema, 2013). For example, two complementary heuristic strategies, each adopted by half of the experimental subjects, could give the impression of mastery of the task, while in reality each participant is only doing part of the required cognitive work. This makes the analysis of individual behaviors crucial. Only once individual strategies have been determined may data be combined to shed light on the nature of learning and hypothesis formation in the species (cf. Fagot, Gullstrand, Kemp, Defilles, & Mekaouche, 2013). Differences within a species, but between groups with different training experience, may also be examined in this way.

Effective analysis of species’ behavioral and cognitive differences may prove useful in experimental design. Theoretically, grammar learning experiments must test a huge number of rule-breaking stimuli in order to rule out all possible alternative hypotheses. In practice this is not possible, and a selected subset of strings must be used as controls, possibly influencing the strategy learned by participants. Usually, different control strings are used for each grammar, but if different grammars could be shown to bias individuals toward specific heuristics, new strings that cannot be learned with that specific heuristic could be incorporated to push acquisition in the direction of the desired strategy (Chen et al., 2014, van Heijningen et al., 2013).

In the current study we examined visual pattern learning. Animal pattern learning experiments to date have mostly focused on the auditory domain (Abe and Watanabe, 2011, Fitch and Hauser, 2004, Gentner et al., 2006, Murphy et al., 2008, Peña et al., 2002, Ravignani et al., 2013, ten Cate and Okanoya, 2012, van Heijningen et al., 2009, Wilson et al., 2015, Wilson et al., 2013, Zuidema, 2013), with only a few studies in other modalities (mostly visual, but also tactile sequence learning (Conway and Christiansen, 2005, Conway and Christiansen, 2006, Herbranson and Shimp, 2008, Ravignani et al., unpublished results, Sonnweber et al., 2015, Stobbe et al., 2012, Westphal-Fitch et al., 2012).

### 1.4 A new analytical framework for animal pattern learning

The present study was focused on the question of how complex patterning rules are individually learned and processed in animal cognitive experiments. Using visual patterns, we tested keas (*Nestor notabilis*, a parrot species) and pigeons (*Columba livia*) on (i) their ability to learn abstract rules (Fig. 1) and (ii) their predisposition to develop individual decision heuristics. Testing animals using a visual operant task is ideal for an analysis of individual heuristics in pattern perception. Visual presentation allows simultaneous presentation of all elements of a string, and/or simultaneous presentation of multiple stimuli, minimizing limitations due to short-term memory constraints which may vary across species. The operant framework provides the large quantities of data needed to reliably uncover individual heuristics.

Together, pigeons and kea constitute a good pair of model species for visual pattern learning experiments. On the one hand, the visual system of both pigeons and kea is highly developed, providing an accurate representation of the three-dimensional visual world, strongly shaped by the requirements of flight (Martin, 2014). On the other hand, pigeons and kea differ in brain size, ecological niche and cognitive performance in tasks requiring social learning and technical intelligence (Huber & Gajdon, 2006), suggesting that they may exhibit significant cognitive differences.

Ten pigeons and ten kea were previously trained (see Methods, and Stobbe et al. (2012) for details) on two widely-used artificial grammar patterns. Five individuals per species were trained on (AB)*^n^*, consisting of a varying number of concatenated AB pairs and five individuals were trained on (A*^n^*B*^n^*), where a variable number of A constituents (henceforth “tiles”) are followed by the same number of B tiles (see Fig. 1). In other words, all birds were trained with simultaneous exposure to (AB)*^n^* and (A*^n^*B*^n^*) patterns, but only responses to one of these patterns resulted in a reward. Birds were tested using touch screens in a two-alternative forced choice (2AFC) paradigm, using pairs of stimuli grouped into five test classes. Each test trial in a test class featured a positive “correct” stimulus, similar to the one reinforced during training, vs. an alternative, that consisted of some non-grammatical combination of As and Bs.

Previous analyses showed that the birds failed to learn the specific patterns that were used to generate these stimuli (Stobbe et al., 2012); the current analysis, based on a much larger dataset featuring all possible negative stimuli, is designed to determine what they *did* learn in these experiments. We analyzed the data using a *model selection* approach to shed light on what drives individual and species differences in performance in pattern learning experiments. Model selection has been a fruitful analytical method in ecology, cognitive science, machine translation, computational linguistics, musicology, and other fields. It contrasts with classical statistics in letting the data “select” the most appropriate model from a pool of candidates, based on their consistency with each model, instead of fitting data points to a single predetermined model.

Our model selection technique addressed four key issues. We aimed to ascertain whether (i) different individual animals used different individual strategies to accept or reject stimuli, (ii) individuals adhered to one particular decision rule or employed a variety of rules, (iii) the particular training grammar would influence the rule heuristics chosen, and (iv) different animal species might adopt different strategies or rule types.

**Fig. 2:**
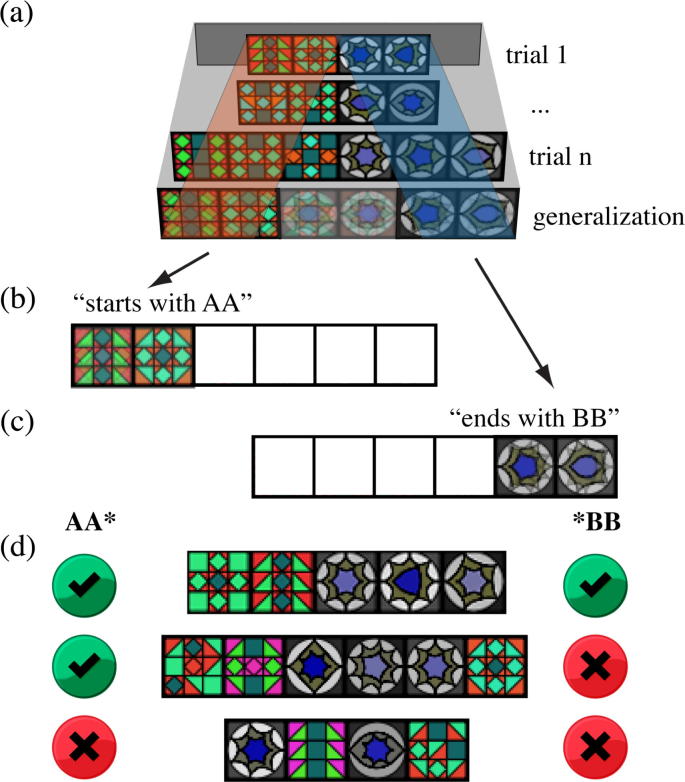
Model selection applied to avian visual pattern learning. The depicted perception, generalization and abstraction steps are crucial in pattern learning experiments in humans and other species. Our model selection approach “reverse-engineers” the outcome of these cognitive processes by comparing the likelihood of many possible hypotheses on how pattern learning occurs in different individuals. (See main text for details.) The generalization phase (a) gives rise to a number of hypothetical alternative strategies (b and c), which are compared using relative likelihood (d).

Fig. 2 provides an overview of the rationale behind our analytical approach. During the training, individuals focus on common properties of reinforced stimuli, e.g., commonalities in patterns of color and shape (Fig. 2a). In particular, all stimuli feature two green/red tiles on the left edge (denoted by the orange rhomboid) and two purple^2^/gray tiles on the right edge (denoted by the blue rhomboid). From these commonalities, the bird can potentially generalize to a number of abstract features, leading to different decision strategies. By analogy, if all trials’ positive stimuli consisted of drawings on glass slides, superimposing all the slides would enhance their common properties and cancel out their differences, as in the generalization phase (Fig. 2a). For example, one possible strategy for a bird trained to choose A*^n^*B*^n^* would be an “AA” primacy rule focusing on the first part of a stimulus to distinguish it from others (Fig. 2b). Alternatively, a bird might adopt a “BB” recency rule and focus on the rightmost part of the stimulus (Fig. 2c). Each strategy will be consistent or inconsistent with some novel test stimuli, leading to its acceptance or rejection in the test phase. A bird’s pattern of choices on these stimuli is compared with the model prediction for every reasonable heuristic (Fig. 2d), and the strategy closest to the prediction is judged to be the most likely. In this example, a primacy rule would be the more likely strategy of the two.

## 2 Materials and methods

### 2.1 Stimuli

Detailed materials and methods of data collection are published in Stobbe et al. (2012). In this section we report the differences between the present study and Stobbe et al. (2012) in detail, and provide a short overview of the methodological aspects common to both studies.

Our experimental stimuli consisted of visual patterns made up of a varying number of complex colored square elements (“tiles”) arranged horizontally. Tiles could contain various visual features, e.g., triangles, squares, circles (see Fig. 1 for an example of two stimuli composed of four tiles each, and two stimuli composed of six tiles each). There were two categories of tiles (As and Bs) that could be easily distinguished by multiple cues within the tiles, such as color and geometry. We created 12 different tiles for each of the two categories (A and B). Visual strings were generated by applying two different production rules, each derived from one of two rule systems (“grammars”). The first grammar, (AB)*^n^*, consisted of a concatenation of a varying number of AB pairs, that is, alternating A and B elements. The second category (A*^n^*B*^n^*), consisted of a varying number of A tiles, followed by the same number of B tiles. Both stimulus classes always began with an “A” tile, ended with a “B” tile and consisted of equal numbers of As and Bs. For both categories, no tile appeared more than once in a stimulus. We used custom scripts in Python and Nodebox (www.nodebox.net) to generate stimuli.

The birds had previously been trained to reliably distinguish A*^n^*B*^n^* and (AB)*^n^* stimuli with *n* = 2 and *n* = 3, and had completed tests that investigated generalization to new colours, grayscale versions and to changes in stimulus orientation. The most crucial generalizations involved novel arrangements of familiar tiles, extensions of the grammar to *n* = 4 and *n* = 5, and “ungrammatical extensions” (foils containing a different number of As and Bs). The results of these tests are published in Stobbe et al. (2012). Here, we report on the decision strategies that the birds used in the following five tests (see Table 2): extensions, foils (two of the eight tests presented and analyzed in Stobbe et al., 2012), string reversals, stimuli that contained A or B tiles exclusively (“pure” stimuli) and a large number of possible permutations (novel data). Extensions were shown to the birds in A*^n^*B*^n^* and (AB)*^n^* pairs, whereas in all the other tests a novel stimulus (from one of the five classes) was paired with a correct example of the grammar for which the birds received a reward during the training (see Supplementary Information for a full list of stimuli):

• *Extensions:* In this test the stimuli were longer (*n* = 4 and *n* = 5) than in training, in order to investigate generalization beyond the previously presented stimulus length. Hence, the stimuli contained either 8 elements (4 A, 4 B) or 10 elements (5 A, 5 B).
• *Nonmatching:* Nonmatching stimuli were obtained by removing one element from grammatical stimuli of length 6 and 8 (corresponding to 3 and 4 tiles per category). Hence, these stimuli had a non-matching number of A- and B-tiles (A*^n^*B*^m^*, where *n* ≠ *m*). To generate these stimuli, we omitted either the first or the last element of a positive stimulus, obtaining strings of the form B(AB)^2^, (AB)^2^A, B(AB)^3^, (AB)^3^A for one group and A^2^B^3^, A^3^B^2^, A^3^B^4^, A^4^B^3^, for the other.
• *Reversals:* In these trials, the A and B tiles were arranged in a reversed order (e.g., BBAA or BABA). In other words, every A tile was substituted for a B tile and vice versa.
• *Pure:* Pure stimuli consisted of 4 or 6 tiles each and were composed of either only A or only B tiles.
• *Permutations:* Combinations of A and B elements were shown that had not previously been used in other tests. These were all possible permutations of A and B tiles which had the same length (4 and 6) as the training stimuli. For instance, the sequence AABABA has length 6 (hence cannot belong to the “extensions” and “non-matching” categories), and is neither a “pure” nor a “reversal” stimulus. Therefore AABABA falls in this last “permutation” category.

### 2.2 Subjects and experimental procedures

#### 2.2.1 Subjects

We compared performance of ten keas (*Nestor notabilis*) and ten pigeons (*Columba livia*) using the same stimuli and grammars. At the onset of the experiment all birds were already familiar with 2AFC experiments on touch screens. For each species, 5 birds were assigned to the A*^n^*B*^n^* group and 5 to the (AB)*^n^* group. Birds of both species were group-housed in outdoor aviaries. Kea resided at the Haidlhof Research Station, Bad Vöslau, (Lower Austria) and received additional food three times a day. Pigeons were housed at the University of Vienna and had free access to water and grit, but did not receive further food apart from occasional additional feeding directly after the experimental sessions and *ad libitum* feeding on weekends. Animal housing and experimental setup followed the Animal Behavior Society Guidelines for the Use of Animals in Research, the legal requirements of Austria and all institutional guidelines.

#### 2.2.2 Materials

For the tests described here, birds had to peck on a 15″ TFT computer screen. Pecks were detected with an infrared touch frame (Carroll Touch, 15″), mounted in front of the screen. The experiment was controlled by CognitionLabLight, version 1.9 (^©^M. Steurer), which also acquired and recorded the data (Steurer, Aust, & Huber, 2012).

All birds were tested individually: keas in an experimental chamber open on one side, pigeons in closed Skinner boxes. Food rewards were 1/8 of a peanut dispensed via a small chute for the keas, and, for the pigeons, a small amount of grain, delivered to the birds by a piston. In both cases, the birds accessed the food directly below the horizontal center of the screen.

#### 2.2.3 Training and test

Two images were presented in a 2AFC procedure. Birds were randomly assigned to one of two groups: for one group pecks on the (AB)*^n^* images were rewarded, for the other group pecks on A*^n^*B*^n^* were rewarded. Birds were first trained to criterion (following the methods and parameters described in Stobbe et al., 2012), and then tested over a series of sessions. Each session consisted of two trial types: rewarded “training” trials and unrewarded “test” trials. In training trials an A*^n^*B*^n^* and an (AB)*^n^* image were shown side by side in fixed positions on a black background. Only choices of the correct stimulus were rewarded. In test trials a regular instance of the positive class (either an A*^n^*B*^n^* or an (AB)*^n^* stimulus, depending on the experimental group) was presented together with a stimulus that violated the rule that defined the positive class. In these test trials, both stimuli were novel; neither choice was rewarded and a touch immediately terminated stimulus presentation. For both training and test trials, stimulus positions (left vs. right) were randomized in every trial.

The birds usually completed one or two sessions (i.e., 40–80 trials in total) per day, depending on individual motivation. Trials were separated by an inter-trial interval of 4 s during which the screen was black. If a 40 trial session was not completed, it was resumed the next day at the point where it had been aborted the day before. Test trials involved stimuli from the five tests classes described above, whereas training trials were pairs of familiar stimuli that were shown in order to maintain both the learned discrimination and the birds’ motivation. Training trials also provided a baseline with which test performance could be compared. In each session, 20% of the trials (8/40) were non-rewarded test trials. The test stimuli were interspersed among rewarded training trials in a semi-random fashion, so that a session never began with a test trial and there was always at least one training trial between test trials.

A correct choice in a training trial resulted in both stimuli disappearing from the screen, playback of a positive feedback sound (600 Hz, 0.5 s) and delivery of a food reward. If the bird pecked on the incorrect image, the stimuli disappeared and a negative feedback sound (200 Hz, 0.5 s) was played, along with 3 s of red-colored screen. The same pair of images was shown again after such failures, and the trial was repeated until the bird eventually pecked on the correct stimulus and received a reward. In probe trials, the bird’s first peck on either of the two stimuli caused the images to disappear, without any feedback, food reward or subsequent correction trial. In both training and test trials, when birds made multiple pecks only the first peck within a stimulus was counted as a choice (and its time and location recorded, see Supplementary Information). Pecks on the screen that were not within a stimulus were recorded, but did not count as choices and were not analyzed.

### 2.3 Statistical analysis

To find out which strategy individuals used to make their choices, an extensive set of possible heuristic strategies was analyzed. Heuristics are defined here as structure-based templates which allow their user to accept or reject a test stimulus. Analysis of strategies is based on the assumption that birds were choosing the reinforced stimulus (alternative assumptions, e.g., avoidance of the non-reinforced stimulus, are considered in the Discussion section). These potential strategies were assigned to four main classes (see Table 1 for details): local, when based on only a few elements, global, if based on the stimulus in its entirety, pattern-matching, when accounting for overall element-wise similarity, and side strategies, if resulting from a bias to peck more frequently on one side.

#### 2.3.1 Local strategies

*Local strategies* are based on substrings of length 2 (i.e., bigrams) appearing at the leftmost edge of the stimulus (primacy rules: AB^*^ and AA^*^), its rightmost side (recency rules: ^*^AB and ^*^BB) or somewhere at any non-edge position (interior rules: +AB+, ^*^BA^*^, +AA^*^ and ^*^BB+). The mathematical notations + and ^*^ stand for any possible sequence of elements extending the string preceding/following it; ^*^ also includes empty sequences, while + must be replaced by at least one element. For instance, ^*^AB+ is a correct notation for strings XY**AB**Z and **AB**XY, but not for XY**AB**). The labels “primacy” and “recency”, and “start” and “end” are used in the sense of the European cultural convention of reading strings from left to right. However, we had no *a priori* prediction that our birds would preferentially parse the strings in one or the other direction.

#### 2.3.2 Global strategies

*Global strategies*, in contrast, allow experimental subjects to make their decisions based on overall visual properties of the stimuli (e.g., transition or non-transition similarity strategies). If subjects focus on visual homogeneity or heterogeneity in the training stimuli, they will continue to apply such a global choice rule in test trials. While the heterogeneous (AB)*^n^* stimuli feature the largest number of transitions (A ↔ B), the more homogeneous A*^n^*B*^n^* strings contain only one AB transition. A successful discrimination could thus be made on the basis of either the absolute number of transitions in a stimulus or the number of transitions as a fraction of its length. Hence, both relative transition strategies (TS and NTS, based on the number of transitions divided by stimulus length in tiles) and their absolute counterparts (aTS and aNTS, based on the number of transitions) were entered in the analysis.

#### 2.3.3 Pattern-matching strategies

*Pattern*-*matching strategies* involve strategies mirroring the rules used to generate the stimuli. Using an (AB)*^n^* rule, stimuli of any length that consist only of AB pairs would be accepted by pattern-matching. In contrast, under an A*^n^*B*^n^* strategy, only those stimuli containing a cluster of A tiles followed by a cluster of B tiles of precisely the same length would be accepted.

The *Partial Block Matching* strategy (BLOCK) captures choice rules that lie halfway between complete pattern matching and local processing and extends the classical Hamming distance (Hamming, 1950) to strings of unequal length. This strategy assumes that a participant will remember the class of shortest stimuli induced during the training (AABB or ABAB, depending on the experimental group) and look for it in the longer test stimuli at any position within the string. If no such match is possible, the participant will choose the stimulus containing the substring that most closely resembles the training string (minimizing the amount of “character” substitutions).

#### 2.3.4 Side strategies

Finally, *side strategies* (left and right bias) capture a lower-level behavior, namely the tendency to peck on one side of the screen irrespective of the stimuli shown. Such side biases often play a role in cognitive experiments, although the possibility of such biases is not always acknowledged in pattern learning research (Herbranson & Shimp, 2008).

#### 2.3.5 Priors and error rates

Individual performance in all tests was evaluated using a model-selection analysis based on maximum likelihood. A uniform distribution was chosen as the prior over strategies (Johnson & Omland, 2004); this neutral choice means that every strategy entered into the model was treated *a priori* as equally likely (Wasserman, 2000, Zucchini, 2000).

For each stimulus pair presented, we determined which perceptual cues were compatible (Bröder and Schiffer, 2003a, Bröder and Schiffer, 2003b) with each of the strategies detailed above, allowing us to predict the choice of a stimulus based on a particular decision strategy.

For each bird, a specific error rate was estimated, based on that individual’s median performance across five test sessions in the reinforced training trials (examples of training patterns are shown in Fig. 1) (Bröder and Schiffer, 2003a, van Heijningen et al., 2009). Stimulus choices were used to calculate log-likelihoods and Akaike weights for each hypothetical strategy (Wagenmakers & Farrell, 2004). When relative likelihoods were close to 1, all strategies up to a cumulative Akaike weight of 0.95 were included in the confidence set (Johnson & Omland, 2004). Statistical analysis and data processing were performed using custom written Python software (www.python.org), following guidelines in Anderson (2008) and Bröder and Schiffer (2003a).

## 3 Results

### 3.1 Overall performance

An analysis of individual performance by test class (using two-tailed binomial tests) revealed some species-dependent and possibly group-dependent differences in performance (Table 2). All birds failed the key test featuring stimuli of non-matching lengths between A and B, but kea succeeded overall in all other tests (*p* < 0.01). The number of pigeons that performed above chance varied for each test and differed between experimental groups.

### 3.2 Individual performance and reaction time

Unsurprisingly, the birds performed worse on novel, unrewarded test strings compared to familiar training stimuli. A Wilcoxon signed-rank test, comparing the overall error rate between training and test trials (Table S1, Supplementary Information) showed a significant difference (*n* = 20, *W* = 1.0, *p* < 0.001, normal approximation). However, the latency in responding was not significantly different between training and test trials. For each bird, test class (as in Table 2), and trial type (training vs. test trials), we calculated the median reaction time. The median latency across conditions (Table S1) was compared between training and test trials for each bird. A Wilcoxon signed-rank test revealed no significant difference (*n* = 20, *W* = 83.5, *p* = 0.469, normal approximation) in the latencies of training and test trials and between correct and incorrect trials (*n* = 20, *W* = 101.0, *p* = 0.881, normal approximation). Thus, latency was not a predictor of certainty or of performance, as neither pigeons nor keas showed a difference in reaction time between training and test trials, or between correct and incorrect trials.

### 3.3 Strategies

**Fig. 3:**
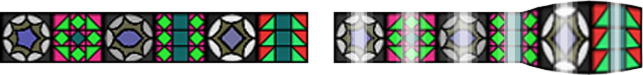
Strategy-based enhancement of stimulus features. During the training, individuals are presented with an experimental stimulus (left), which is processed as described in Fig. 1, producing a cognitive representation based on an individual heuristic (right). In this particular example (based on the analysis of pigeon P15), decisions on whether to peck on the stimulus are based on its rightmost part (inflated in the figure), and the transitions between contiguous squares (enhanced).

Differences in strategies used to solve the tasks were found at three levels: individual, species and experimental group. In general (see Table 3, Table 4 for details), the keas’ choices in the (AB)*^n^* group were all consistent with the same local strategy (^∗^BA^∗^, corresponding to accepting strings which contained a “BA” bigram), while pigeons in the (AB)*^n^* group employed a greater variety of strategies (between two and five different strategies per bird). All pigeons showed some evidence of having employed the global transition similarity strategy (in its relative, TS, or absolute variant, aTS). Additionally, all pigeons in this group used a pattern-matching strategy. For one pigeon (P15), the (AB)*^n^* pattern strategy was the second ranked in order of descending likelihood (corresponding to the second most likely strategy, see Fig. 3). For all other pigeons in the group, the BLOCK strategy was among the most likely strategies.

Turning to the A*^n^*B*^n^* grammar, all kea in this group employed an “edge” strategy, basing their choice behavior on either the last (^∗^BB) or the first two tiles (AA^∗^). Each bird consistently followed only one of the two strategies and did not switch between them. One individual kea (K6) additionally made use of the BLOCK sub-pattern matching rule. In contrast, individual pigeons in the A*^n^*B*^n^* experimental group made use of a variable number of strategies, ranging from one to six. Unlike in the (AB)*^n^* group where the global strategy TS dominated, local strategies were often the most likely for pigeons in the A*^n^*B*^n^* group. In some cases, these were complemented by strategies based on a side bias, by the global TS strategy or by the pattern-matching BLOCK rule. The intended generative rule (i.e., A*^n^*B*^n^* or (AB)*^n^*) was not the most likely strategy for any of the birds.

We calculated the overall performance for each strategy, as a grand average of birds’ success rate (in reinforced trials) weighted by the Akaike weight for each bird and strategy. The strategies associated with the highest performance (>80%) were ^∗^BB, AA^∗^ and ^∗^BA^∗^. Strategies associated with the worst performances (<65%) were aTS/aNTS (pooled together), ^∗^BB+, ^∗^AB and (AB)*^n^*.

### 3.4 Pecking location

To make their choice, birds pecked on a touch screen anywhere within the stimulus. To determine whether peck location was tied to strategy, we analyzed each individual’s pecking location within a chosen image. We found that individual strategy choice, as determined by our model selection approach, was mirrored by the pecking location. Thus peck location appears to provide a behavioral “readout” for individual strategy.

**Fig. 4:**
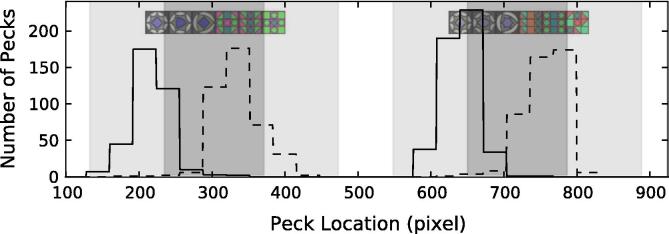
Pecking locations of two keas, K11 (unbroken line), using a recency strategy, and K4 (dashed), employing a primacy strategy. The shortest horizontal “step” corresponded to 32 pixels on the screen (approximately 9 mm) and approximates the width of a single tile. Dark and light gray areas denote the minimum and maximum stimulus size, respectively. The vertical axis measures the total number of pecks per bird within the corresponding 32 pixel window.

**Fig. 5:**
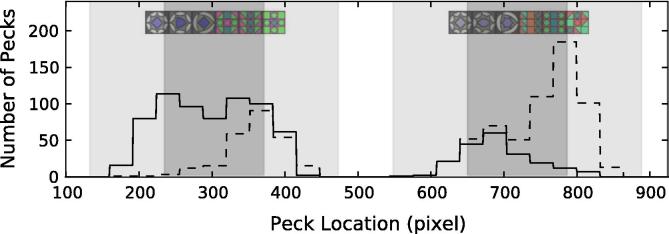
Pecking locations of two pigeons. P19 (unbroken line) used a mix of five strategies. P16 (dashed) used a combination of recency and side bias strategies.

Fig. 4, Fig. 5 show the frequency of pecking at different horizontal locations on the screen. The frequencies shown in the figures are the sum of all pecks within a 32 pixel window, a rough approximation of the average tile size. (32 pixels is an even divisor of 1024, the screen horizontal resolution, lying between 28 and 34 pixels, the two possible horizontal widths of tiles.) Fig. 4 shows how one kea (K11, unbroken line) who used a primacy rule, pecked on the left side of each stimulus, while the other (K4, dashed line) who used a recency rule pecked on the right side. Fig. 5 shows pecking frequencies of P19 (unbroken line, a bird that used 5 different strategies) and P16 (dashed, that used a mix of recency and side bias strategies). P16 pecked relatively more often on the right side than on the left of each stimulus (recency rule) and relatively more frequently on the right side of the screen, rather than the left (side bias). Individual differences were also present in the distribution of pecks. (see Fig. S1 in Supplementary Information, showing the distribution and variability in pecking location for each bird and side of the screen.)

### 3.5 Transition similarities and error patterns

**Fig. 6:**
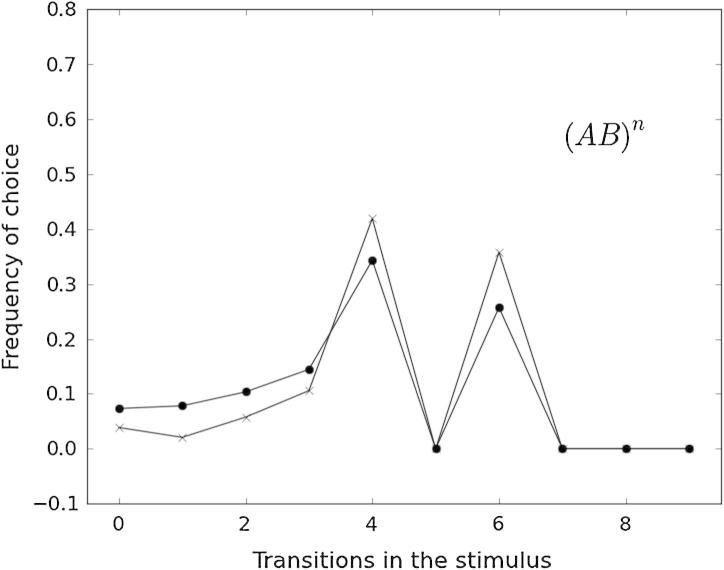
Number of transitions in the (incorrectly) chosen stimulus (*x*-axis) and frequency of choice for pigeons (dots) and kea (crosses) of the (AB)*^n^* group.

**Fig. 7:**
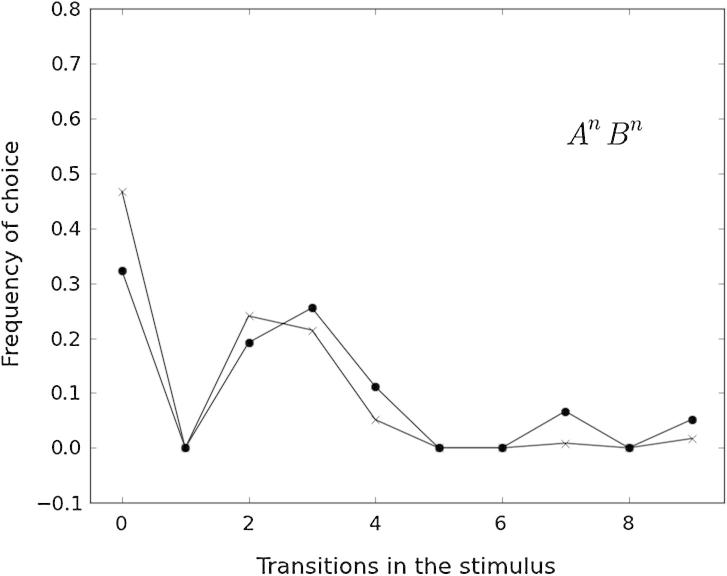
Number of transitions in the (incorrectly) chosen stimulus (*x*-axis) and frequency of choice for pigeons (dots) and kea (crosses) of the A*^n^*B*^n^* group. Only misclassifications due to a different number of absolute transitions are included.

**Fig. 8:**
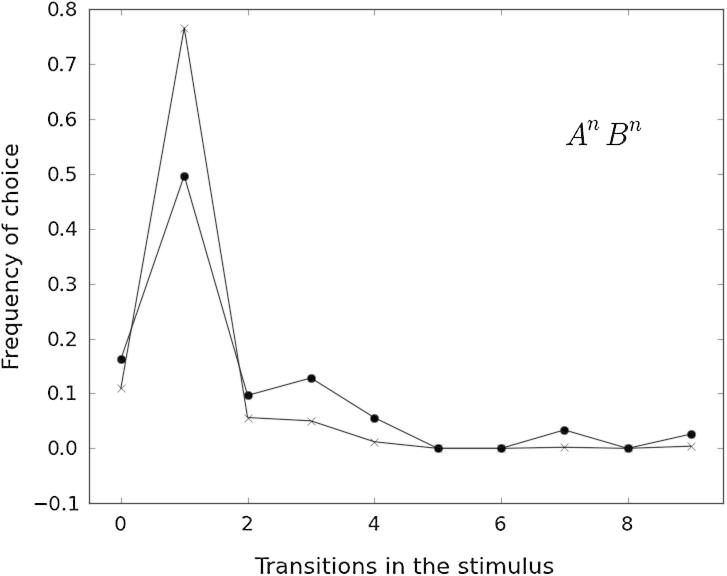
Number of transitions in the (incorrectly) chosen stimulus (*x*-axis) and frequency of choice for pigeons (dots) and kea (crosses) of the A*^n^*B*^n^* group. Misclassifications due to a different number of either absolute transitions or relative transitions are included.

Transition similarity rules appear to partially explain overall error patterns. Fig. 6, Fig. 7, Fig. 8 show the frequency of *incorrectly* chosen test patterns as a function of the number of A ↔ B transitions in them. To analyze misclassifications based on global transition properties of the stimuli, we only included trials where the two stimuli had a different (relative or absolute) number of transitions. (For a discussion on relative vs. absolute transition rules, see Section 2.3.2.)

Fig. 6 refers to the experimental group (AB)*^n^*, for which birds adopting the transition-similarity rule would choose the stimulus with the largest absolute number of transitions. Even though entries between 0 and 5 transitions show an increasing trend, Kendall’s Tau correlations were not significant for either species (*p* > 0.29). This may be driven by the sharp contrast between frequencies of 4, 5 and 6 transitions, probably due to the fact that the S+ for this group contained exactly 5 transitions. (Analyses including relative transitions for this group produced identical results.)

Fig. 7 shows error patterns of birds in the A*^n^*B*^n^* group conditional on stimuli having a different *absolute* number of transitions (Kendall’s Tau correlations were not significant for either species, *p* > 0.10). Fig. 8 shows error frequencies from the same birds, including also trials with different *relative* number of transitions (Kendall’s Tau: *T* = −0.64, *p* < 0.01 for both species). There is a major difference between the two: incorrectly choosing AAABB over AABB can be accounted for by (relative) TS and NTS strategies (because the stimuli have a different number of elements) but not by (absolute) aTS and aNTS strategies (because the number of transitions is 1 in both cases, so this strategy cannot be used to discriminate this pair of stimuli). Fig. 7, Fig. 8 together suggest two potentially complementary hypotheses concerning the nature of errors in the A*^n^*B*^n^* group. In terms of absolute transitions, birds misclassified stimuli with 4 transitions or less (Fig. 7). In particular, almost half of kea’s erroneous choices contained 0 transitions: these are the pure (all A or all B) stimuli, which are likely to be chosen by edge-based strategies (primacy or recency). In terms of relative transition similarity, most misclassifications contained 1 transition (Fig. 8). These are often the non-matching stimuli, composed of an A block and B block of unequal length. Choice of these stimuli is consistent with both local and global strategies.

## 4 Discussion and conclusions

### 4.1 Differences in number, type and heterogeneity of strategies

Visual perception and discrimination are cornerstones of comparative cognitive research between humans and other animals. This study presents a novel approach to analyzing individual-level behavior, focused on uncovering cognitive heuristics and sources of individual variation in visual pattern perception. Our model selection approach revealed differences in the number, type and heterogeneity of decision rules adopted in two bird species, both at the individual and species level.

The main differences were seen between species rather than between experimental groups. Overall, keas tended to focus on a single local strategy, while pigeons typically employed an idiosyncratic mix of several strategies of different types (global, local, bias, and pattern-matching strategies). Keas used one, or at most two, *local* strategies, regardless of training pattern. Pigeons in both groups used many mixed strategies, some of which were global or pattern-matching.

Analysis of error patterns provided evidence of between-species’ similarities and within-species differences influenced by experimental group. Analysis of error patterns for the (AB)*^n^* group supports the hypothesis that individuals from both species were focusing on the stimulus’ interior elements. A more uniform error distribution for pigeons than for keas dovetails with the heterogeneous strategy set found in the former species. Error patterns of both species in the A*^n^*B*^n^* group can mostly be explained by birds choosing stimuli containing 0, 2, 3 or 4 transitions (peaks in Fig. 7). Birds employing either local or global strategies could exhibit this pattern. The most frequent error type (choosing 0 transitions or all-A or all-B stimuli) might result from using a local strategy (AA^∗^ or ^∗^BB), used by 9 out of 10 birds (Table 4). Fig. 8 shows a more uniform distribution for pigeons than for kea. The peak at one transition is probably a result of kea choosing a mismatched A*^n^*B*^m^* stimulus with an extra A at the beginning or B at the end (i.e., *n* ≠ *m*). The broader peak for pigeons might result from these animals using mainly local strategies and complementing them with global ones to a lesser extent. Overall, the analyses of error patterns seem to support and extend the results obtained by model selection.

### 4.2 Learning rules or memorizing chunks?

Neither of the exact grammars used to generate the training stimuli was adopted (Stobbe et al., 2012) as the most likely strategy for any pigeon (although a single pigeon, trained on instances of (AB)*^n^*, may have actually adopted this abstract pattern as *one* of its decision rules). Using only single bigram-based strategies, keas were able to attain very high levels of performance in all tests, except those featuring a non-matching number of As and Bs. These keas, and some pigeons in the A*^n^*B*^n^* group, behaved similarly to occasional humans who fail to learn the abstract generating pattern in similar experiments (Hochmann et al., 2008, Zimmerer et al., 2011): in such cases, the incorrectly chosen stimuli with highest frequency contain one transition, that is, correspond to A*^n^*B*^m^* or B*^m^*A*^n^*. Although most humans trained on this grammar reject mismatches of *n* and *m* (Stobbe et al., 2012), showing that they noted and generalized the match between A and B chunks, a single-transition decision rule is sometimes found in a subset of human participants (Zimmerer et al., 2011). This supports the idea that a model selection approach, focused on individual performance in humans (Zuidema, 2013) might reveal further interesting similarities between humans and other species.

Our findings confirm some results of previous grammar learning studies in pigeons (Herbranson & Shimp, 2008). Pigeons in Herbranson and Shimp (2008) had a left/right bias toward one of the strings, analogous to a side bias, which explained some of our pigeons’ choices. Moreover, pigeons seem to have used two possible strategies to accomplish the task (Herbranson & Shimp, 2008): training-based (“memorize chunks”) vs. grammar-based (“learn underlying rules”). Our results seem to point toward related “training-based” strategies: with our training, pigeons memorized chunks rather than rule-based properties of the stimuli (see Kürten, De Vries, Kowal, Zwitserlood, and Flöel (2012) for a human perspective).

### 4.3 Within- and between-species variation

Our results do, however, differ from previous results from pigeons and keas in some other respects. Previous cognitive studies have characterized kea as explorative, seeking optimal solutions by trial and error. Our analyses, by contrast, suggested that most keas chose a single strategy and stuck with it. However, kea might have first explored multiple options before lighting upon a satisfactory strategy during the training sessions, and then subsequently adhered to it, producing a stable behavior in the later test sessions analyzed here.

In pigeons, a local bias has repeatedly been observed, which has become known as a “local precedence effect” (Cavoto and Cook, 2001, Cerella, 1980, Gibson et al., 2005), in contrast to the global bias often found in humans (Navon, 1977). However, solid evidence has emerged that this “pigeon: local; humans: global” generalization may be an oversimplification. Indeed, in humans (Pomerantz, 1983) as well as in pigeons (Aust and Huber, 2001, Cook, 1992, Goto et al., 2004; E. A. Wasserman, Kirkpatrick-Steger, Van Hamme, & Biederman, 1993), either local or global information (or both) can influence performance, and attention can be flexibly shifted between parts and wholes. Previous work examined (i) whether structural, syntactic-like rules have an influence on the level of processing (global vs. local) and (ii) where different species stand along this global–local continuum. Our results support a “shifting” hypothesis for pigeons: both local and global information were used in our pattern learning experiments. The strategies that our pigeons adopted were not as local as expected: much of their behavior could be explained through a mix of global and other decision rules (for instance, see pigeon P19, Fig. 5), but they nonetheless performed poorly using such “mixed strategies”. Keas, in contrast, appeared to employ *only* local information when choosing a stimulus, and in general, outperformed pigeons in both training trials and test trials. Because high performance, test species, and adoption of local strategies are all inter-related, it is difficult to establish any clear causal relation among these different factors based on the current data.

The species differences we observed might be due to differences in ecological backgrounds (Mettke-Hofmann, Winkler, & Leisler, 2002). Keas are a New Zealand parrot species famed for their highly-developed technical cognition and visual discrimination abilities (Auersperg et al., 2009, Auersperg et al., 2011, Huber and Gajdon, 2006, O’Hara, 2011, Schloegl et al., 2009), suggesting the existence of good abstract pattern recognition in this species. For instance, seasonally foraging on about 200 different plants and cryptic plant parts requires these birds to learn specific natural patterns (Brejaart, 1994). Kea are flexible foragers and problem-solvers (Diamond & Bond, 1991): In their natural environment, and when tested on physical cognition tasks, kea often quickly come up with novel, insightful solutions without the need of a trial-and-error phase (Huber and Gajdon, 2006, Miyata et al., 2011). This may also have happened in our experiments.

Pigeons’ mixture of small brain size and high visual competence makes them an interesting model species for comparative research (Cook, 1992). Their vision is well studied, and pigeons excel relative to humans in some visual capacities. Pigeons seem able to cope with sophisticated visual tasks, as long as they can find a superficial perceptual anchor for their decisions. This often involves using perceptual rules and similarities, when present, to provide a response. However, pigeons often fail in abstract tasks that require categorization beyond a superficial perceptual level (i.e., recognizing abstract relations, although pigeons can learn some artificial grammars: see e.g., Herbranson & Shimp, 2008). But both the limits of pigeons’ abstract categorization abilities and the precise cognitive mechanisms used to accomplish visual tasks remain a matter of ongoing debate. Our pigeon subjects, in contrast to keas, never actually found a good working approach to the specific problem posed, and some of them kept trying alternative possibilities.

It is also worth noting that the Akaike weights reflect statistical confidence that a given strategy was employed. Many selected strategies with corresponding low Akaike weights could either mean that the bird was behaving erratically, or that the analysis is not fully confident of any single strategy, but rather provides a set of them as best guesses. Also, a bird switching between fixed strategies over time would appear in this framework as a bird maintaining one mixed-strategy behavior over time. We did do additional analyses on subsamples of the data to test this switching vs. mixed strategy hypotheses (see Supplementary Information). It seems that both kea groups, and probably the (AB)*^n^* pigeon group, started the experiments with a larger strategy set and converged toward a smaller strategy set toward the end of the experiment. Pigeons in the A*^n^*B*^n^* group kept a fixed core of strategies throughout the testing, and replaced some other strategies over time. This suggests that most birds had already found some of their final strategies at the beginning of the experiment.

An additional source of the observed species differences could involve initial learning performance differences. Although both species reached criterion (corresponding to 70% or more first correct choices per session in six consecutive sessions, Stobbe et al., 2012, pg. 1999) during the initial training, the performance asymptote reached by pigeons was much lower than by kea (as suggested by error rates between 1% and 5% for keas, and between 19% and 40% for pigeons in repetition training trials, see Table S1 in Supplementary Information).

### 4.4 Individual strategies

Our results confirm and extend previous work applying a model selection approach to auditory pattern perception that indicated the use of local strategies (primacy, recency and interior rules) by zebra finches (van Heijningen et al., 2009). Here we show that similar local strategies were used by two further unrelated bird species, even when tested in a different modality (visual instead of auditory) and setup (2AFC here, instead of go/no-go). However, any direct comparisons of zebra finches, pigeons and kea need to take into account methodological and analytical differences between these studies. Our stimuli were always presented in pairs, and our analysis thus includes an effect of context on decision-making (the appearance and structure of the two stimuli relative to each other). Such context effects in animal pattern learning experiments are well known (Herbranson & Shimp, 2008).

Keeping this methodological difference in mind, we found that some individuals used novel non-local strategies, not analyzed in the van Heijningen study. By including global and pattern-matching strategies, our results suggest that individual birds’ and species’ decision rules may occupy a multidimensional strategy space. At two extremes of this ideal space we would find purely local or global strategies. Our local strategies were based on bigrams (the shortest possible stimuli parts allowing an effective decision rule, since strategies based on single tiles would not have distinguished even the training stimuli). Keas in both experimental groups seem to have focused exclusively on such bigram strategies. Global strategies, in contrast, were based on overall visual properties of the stimulus with respect to transition structure between tiles. Pigeons’ choices in both experimental groups, and some of the error patterns in the A*^n^*B*^n^* group, were best explained by the adoption of such global rules. Global strategies corresponded to visual homogeneity (few transitions) or heterogeneity (several transitions), and could be easily reformulated in terms of entropy-based decisions (see for instance (Pothos, 2010)). Between these two extremes, our Hamming distance-based BLOCK rule captures an intermediate level of information processing: although the entire stimulus is not considered globally, its subparts must resemble a predetermined overall template. Some pigeons appeared to have employed such an intermediate-level strategy, suggesting that pigeons tested in other visual experiments might employ strategies lying between local and global (like BLOCK) rather than a mixture of global and local strategies *per se*. BLOCK was, in fact, the most likely strategy for two of ten tested pigeons. Given the importance of the long-running local/global debate in avian visual cognition, we suggest that new insights could be gained by using BLOCK and related metrics in analysis of data from other pigeon experiments. Rather than providing an alternative to previous theories, the metrics and model selection approach we propose could integrate and complement previous theoretical frameworks, such as the Modified Feature Theory of visual categorization (Huber & Aust, 2006).

Finally, independent of the stimulus choice itself, assessment of individual pecking locations provided an additional source of confirmation for the individually variable heuristics inferred using the model selection process, showing agreement between spatial information in the behavioral data, and supporting statistical inferences about the underlying cognitive processes. Pecking location thus seems to provide an online behavioral read-out of an individual bird’s current strategy and perceptual focus: a fact that could be employed during training to break bad habits before they are formed.

### 4.5 Future work

It is important to note that, although we utilized a large number of possible strategies in our initial model set, the set is not exhaustive. An even larger (in principle infinite) number of strategies could have been tested. Some strategies have been excluded for implausibility (e.g., reject all stimuli with a prime number of Bs), or because they were unlikely to have developed during training (e.g., accept only pure-A stimuli). Several more complex and composite strategies (e.g., accept stimuli starting with an AB, containing few transitions and possibly displayed on the left side of the screen) did not need to be explicitly represented because our model-fitting procedure could still output them as a weighted set composed of simpler strategies (in this example, startAB, low TS and left bias). However, some plausible strategies were not entered in the analyses. Our model set features local (i.e., bigram), global and pattern-matching strategies, but it does not contain larger *n*-gram (e.g., trigram) strategies. Although heuristics captured by *n*-grams and pattern matching partially overlap, larger *n*-grams could have entered some of the birds’ Akaike set if evaluated by the model. Alternatively, in our 2AFC paradigm some of the birds could have followed avoidance-based rules instead of acceptance-based strategies. In other words, birds in the (AB)*^n^* group could follow e.g., an “avoid a startAA stimulus” (the opposite of the “accept startAA” from A*^n^*B*^n^*), instead of “accept a startAB stimulus”. More generally, using logical negation, all strategies prescribing stimulus *acceptance* in the (AB)*^n^* group could be used as stimulus *avoidance* strategies for the A*^n^*B*^n^* group, and vice versa. Future research might enlarge the strategy set evaluated, e.g., including *n*-gram and/or avoidance-based strategies in the analyses, thus potentially providing an even more nuanced picture of individual pattern learning.

Our results help clarify the ongoing debate concerning which non-human animal species can master the A*^n^*B*^n^* grammar, a pattern used as a proxy for the supra-regular cognitive abilities underlying language. In light of our own and previous related findings, it is crucial that whenever possible future animal pattern learning experiments analyze individual participants’ strategies, since merged group data can be misleading. The model selection approach we use here seems particularly well suited for experiments testing participants on a large number of trials and in an operant setup in the visual or auditory modalities (as opposed to habituation/discrimination methods, which produce far less data (Ravignani and Fitch, 2012, ten Cate and Okanoya, 2012).

### 4.6 Conclusions

Artificial grammar learning is a topic of active research, with recent findings informing language, music and visual cognition (Christiansen et al., 2010, Conway et al., 2011, Culbertson and Adger, 2014, Fitch and Friederici, 2012, François and Schön, 2014, Loui et al., 2010, Ravignani et al., 2013, Rohrmeier et al., 2012, Rohrmeier et al., 2011, Smith and Wonnacott, 2010, Sonnweber et al., 2015, Spierings and ten Cate, 2014, ten Cate, 2014, Westphal-Fitch et al., 2012, Wilson et al., 2015, Zimmerer et al., 2014). A model-selection approach to pattern learning and animal cognition can inform and refine not only the scientific inferences we draw from such experiments, but also future experimental designs. This is true both for comparative pattern learning studies and comparative cognition research in general.

Our results suggest that different species may adopt radically different strategies to make sense of incoming sensory patterns, and to infer their structures and possibly compress the relevant information. That is, an approach that seems simple or “natural” to one species may not be so obvious to another. Moreover, our findings emphasize the need to take individual behavior into account before reaching conclusions about species variability. Only if species differences reflect consistent individual behavior in multiple individuals (as for our keas) does it seem appropriate to compare behavioral data with information about neural or genetic differences between species. This approach will enrich the more general research program attempting to uncover how neural structures map onto cognitive abilities and behavioral strategies both on an individual and species level (Chittka et al., 2012, Fitch, 2014, Ravignani et al., 2014).

## Figures and Tables

**Table 1:** Main strategies entered into the analysis. Note that each strategy in the first experimental group has its equivalent in the second. AB^∗^ corresponds to AA^∗^ (primacy rules) and ^∗^AB corresponds to ^∗^BB (recency rules); +AB+ and ^∗^BA^∗^ correspond to +AA^∗^ and ^∗^BB+ (interior rules); TS (transition similarity) is the inverse of NTS (non-transition similarity). The last column includes the corresponding regular expression in formal language theory terms (Jäger and Rogers, 2012, Sipser, 2006); the symbol # denotes the absence of a possible regular expression for that strategy.

Group	Rule name	Conditions for stimulus acceptance (“accept stimulus which…”)	Regular expression
**(AB)*^n^***	startAB	starts with the substring AB	AB^∗^
endAB	ends with the substring AB	^∗^AB
interiorAB	contains AB at any non-edge position	+AB+
interiorBA	contains BA anywhere	^∗^BA^∗^
TS	resembles the alternation in the training stimuli (by maximizing the number of transitions between A and B tiles, and vice versa)	#
(AB)*^n^*	consists of any non-zero number of AB pairs	(AB)+

**A*^n^*B*^n^***	startAA	starts with the substring AA	AA^∗^
endBB	ends with the substring BB	^∗^BB
interiorAA	contains AA at any position (except the beginning)	+AA^∗^
interiorBB	contains BB at any position (except the end)	^∗^BB+
NTS	resembles the visual homogeneity in the training stimuli (by minimizing the number of transitions between A and B tiles, and vice versa)	#
A*^n^*B*^n^*	contains an equal number of A and B, and all A tiles precede all B tiles	#

**Both**	RR	is on the right	#
LL	is on the left	#

**Table 2:** Individual performance of the two tested species in all test classes. Shown are the numbers of individuals in a subgroup that significantly mastered a particular stimulus class (binomial test, *p* < 0.01) relative to the total number of individuals (5 for each subgroup). In parentheses, the number of non-reinforced trials used for the calculation (because of a technical problem, one pigeon (P12) had only 272 novel non-reinforced test trials in the *non*-*matching* test). The 320 stimuli for the non-matching test class break down into four sets of 80 stimuli: 3A2B, 2A3B, 4A3B and 3A4B. All possible remaining permutations after the four test classes amounted to *N* = 216.

Test (*N*)	Pigeons		Kea	
(AB)*^n^*	A*^n^*B*^n^*	(AB)*^n^*	A*^n^*B*^n^*
*Extensions (80)*	3	4	5	5
*Nonmatching (320)*	0	0	0	0
*Reversals (120)*	0	1	5	5
*Pure (120)*	5	1	5	5
*Permutations (216)*	1	2	5	5

**Table 3:** Most likely strategies used by kea and pigeons in the (AB)*^n^* group when choosing between two stimuli. Each cell contains the corresponding Akaike weight (multiplied by 100 and truncated), up to a cumulative Akaike weight ⩾0.95. The likelier a strategy, the higher its Akaike weight. Akaike weights in bold denote the highest value for that bird, and correspond to the most likely strategy. For a description of each strategy, see Table 1.

Strategy	Species/ID									
Kea					Pigeon				
K3	K5	K7	K9	K14	P12	P13	P14	P15	P22
**AB**^∗^								4		
^∗^**AB**						14		6	25	
**+AB+**						**33**				
^∗^**BA**^∗^	**99**	**99**	**99**	**99**	**99**		12			
**TS/aTS**						28	**57**	37	**41**	45
**(AB)*^n^***									29	
**BLOCK**						13	28	**50**		**49**
**LL**						7				

**Table 4:** Most likely strategies used by kea and pigeons in the A*^n^*B*^n^* group when choosing between two stimuli. See Table 3 caption for details.

Strategy	Species/ID									
Kea					Pigeon				
K2	K4	K6	K10	K11	P16	P17	P18	P19	P20
**AA**^∗^					**99**	4	16		10	
^∗^**BB**	**99**	**99**	**76**	**99**		32	16	**84**	10	
**+AA**^∗^							**29**		**33**	**98**
^∗^**BB+**							8		29	
**NTS/aNTS**									13	
**A*^n^*B*^n^***										
**BLOCK**			23				24	15		
**RR**						**62**	4			

## Footnotes

2 For interpretation of color in Fig. 2, the reader is referred to the web version of this article.
