# Supplementary material for: More than one way to see it: Individual heuristics in avian visual computation
Source: Cognition. 2015 Oct;143:13–24. doi: 10.1016/j.cognition.2015.05.021 (PMC4710635; doi:10.1016/j.cognition.2015.05.021)
Supplement: Supplementary data 1 [file mmc1.doc]

**More than one way to see it: Individual heuristics in avian visual computation**

**Supplementary Information**

Andrea Ravignani, Gesche Westphal-Fitch, Ulrike Aust, Martin M. Schlumpp, W. Tecumseh Fitch

**S1. Error rates and reaction times**

| **Group** | **Species** | **ID** | **Error rate (%)** | | **Reaction time (msec)** | |
| --- | --- | --- | --- | --- | --- | --- |
|  |  |  | **training** | **test** | **training** | **test** |
| **(AB)^n^** | **Kea** | K3 | 4 | 7 | 661.0 | 650.0 |
|  |  | K5 | 2 | 10 | 1583.0 | 2153.0 |
|  |  | K7 | 1 | 13 | 912.0 | 931.0 |
|  |  | K9 | 2 | 12 | 1001.0 | 1112.0 |
|  |  | K14 | 5 | 19 | 721.0 | 711.0 |
|  | **Pigeon** | P12 | 37 | 44 | 781.0 | 706.0 |
|  |  | P13 | 28 | 37 | 802.0 | 821.0 |
|  |  | P14 | 35 | 43 | 831.0 | 912.0 |
|  |  | P15 | 39 | 38 | 801.0 | 801.0 |
|  |  | P22 | 31 | 42 | 1092.0 | 1112.0 |
| **A^n^B^n^** | **Kea** | K2 | 2 | 12 | 621.0 | 611.0 |
|  |  | K4 | 2 | 10 | 731.0 | 761.0 |
|  |  | K6 | 4 | 13 | 581.0 | 611.0 |
|  |  | K10 | 1 | 11 | 571.0 | 651.0 |
|  |  | K11 | 2 | 7 | 691.0 | 851.0 |
|  | **Pigeon** | P16 | 29 | 40 | 841.0 | 761.0 |
|  |  | P17 | 40 | 49 | 806.0 | 611.0 |
|  |  | P18 | 19 | 28 | 1271.0 | 1182.0 |
|  |  | P19 | 38 | 46 | 711.0 | 651.0 |
|  |  | P20 | 33 | 49 | 1231.0 | 1130.5 |

Table S1. Error rate (as percentage of incorrect choices) and median reaction time (from presentation of the stimulus till valid peck on the screen) for training trials and test trials are shown by species, experimental group and individual. Test trials are unrewarded novel patterns interspersed with training trials; (repetition) training trials are reinforced novel stimuli (i.e. tile combinations), but structurally identical to the initial training patterns. Error rates are calculated as percentage of cases in which the bird did not peck on the training S+ (training column) or its generalization as presented in the test trials (probes column).

**S2. Distribution and variability in pecking location for each bird and side of the screen.**

Figure S1: Box-plots of pecking locations (depicted on the vertical axis) for all experimental groups (left of the central dashed gray line: (AB)^n^; right of the central gray line: A^n^B^n^) and birds (K: kea; P: pigeon).

**S3. Comparison between subsets of strategies**

The model selection results (section 3.3) suggest that kea usually stick to one strategy, while pigeons exhibit a larger and more heterogeneous strategy set. Pigeons could have, in principle, either used a large set of strategies throughout the whole testing period or switched between smaller subsets of strategies. To ascertain this, we repeated the whole analysis in 3.3 applied to only the first and second test periods. First and second periods were defined as the first 400 versus the last 456 test trials (so not to split test classes, see Table 2).

For each individual bird and period, we obtained a strategy set (including Akaike weights). For each bird, we counted the number of strategies in the 95% Akaike set and matched them between periods, so to obtain the number of strategies used in both periods. A Wilcoxon signed rank test (exact, two-tailed) comparing the absolute number of strategies between the first and the second period showed a significant decrease for kea (n=10, W=55.0, p<.01) but not for pigeons (n=10, W=32.5, p=.28).

|  |  | K3 | K5 | K7 | K9 | K14 | P12 | P13 | P14 | P15 | P22 |
| --- | --- | --- | --- | --- | --- | --- | --- | --- | --- | --- | --- |
| Percentage overlap | | 50 | 100 | 100 | 100 | 50 | 100 | 100 | 50 | 80 | 75 |
| Delta-switch coefficient | | 0 | 0 | 0 | 0 | 0 | -0.8 | 0 | 0 | 0 | 0 |

Table S2. Percentage overlap and “delta-switch coefficient” between strategies in the Akaike set for birds in the (AB)^n^ group (see main text for details).

|  |  | K2 | K4 | K10 | K6 | K11 | P16 | P17 | P18 | P19 | P20 |
| --- | --- | --- | --- | --- | --- | --- | --- | --- | --- | --- | --- |
| Percentage overlap | | 100 | 100 | 100 | 100 | 100 | 100 | 83 | 100 | 100 | 0 |
| Delta-switch coefficient | | 0 | 0 | 0 | -0.5 | 0 | -0.33 | 0.17 | -0.50 | 0 | 1 |

Table S3. Percentage overlap and “delta-switch coefficient” between strategies in the Akaike set for birds in the A^n^B^n^ group (see main text for details).

Based on strategy sets from the first, second and overall periods, we calculated a percentage overlap (Table S2, S3), measuring the commonalities of strategy sets between periods. This was calculated as the percentage of the common strategies between periods over the maximum possible common strategies (equal to the minimum number of strategies between the two periods). A percentage close to 100% suggest that strategy sets overlap completely, or that one is contained in the other. Both species and experimental groups showed good degrees of overlap (Table S2, S3). More pigeons than kea however exhibited only partial overlap (less than 100%).

We also matched each period strategy to the “overall” strategies (obtained in 3.3), counting how many of the overall strategies were already present in the first or second period. The delta-switch coefficient (Table S2, S3) measures the extent to which strategies discovered in the first and second period can be found in the overall set of strategies. The coefficient is calculated by subtracting the number of early (overall strategies already present in the first period) from the number of late strategies (overall strategies found in the second period), divided by the number of overall strategies. When early and late strategies coincide, the coefficient is 0, suggesting little or no variation between periods. A coefficient close to 1 suggests that most of the overall strategies were late strategies. A coefficient approaching -1 suggests that most overall strategies were discovered early in testing. Almost all kea and pigeons in the (AB)^n^ group showed no switch (coefficient equal to 0), suggesting that their overall strategy was present in both periods. Among all combinations of species and experimental groups, A^n^B^n^ pigeons exhibit the largest amount of switches, suggesting a search for an effective strategy over time. The dynamics of this search are however unclear: Two birds have a positive coefficient, two a negative coefficient and one bird shows no switches.

Taking into account number of strategies, percentage overlap and switch coefficients, we suggest that most of the birds had already adopted their final strategy (or strategies) in the first period. After the first period, kea – and probably (AB)^n^ pigeons - converged towards a smaller strategy set in the second period. Pigeons instead replaced some of their strategies over time, and the A^n^B^n^ pigeon group shows heterogeneous individual tendencies in converging to the final strategy set: equal numbers of animals found their final strategies early or late.

**S4. List of stimuli tested against S+ stimuli**

**A^n^B^n^ group**

*Extensions*

ABABABAB

ABABABABAB

*Foils*

AABBB

AAABB

AAABBBB

AAAABBB

*Reversals*

BBAA

BBBAAA

*PureA / PureB*

AAAA

AAAAAA

BBBB

BBBBBB

*Permutations*

ABBA

BAAB

AABABB

AABBAB

AABBBA

ABAABB

ABABBA

ABBAAB

ABBABA

ABBBAA

BAAABB

BAABAB

BAABBA

BABAAB

BABBAA

BBAAAB

BBAABA

BBABAA

**(AB)^n^ group**

*Extensions*

AAAABBBB

AAAAABBBBB

*Foils*

BABAB

ABABA

BABABAB

ABABABA

*Reversals*

BABA

BABABA

*PureA / PureB*

AAAA

AAAAAA

BBBB

BBBBBB

*Permutations*

ABBA

BAAB

AABABB

AABBAB

AABBBA

ABAABB

ABABBA

ABBAAB

ABBABA

ABBBAA

BAAABB

BAABAB

BAABBA

BABAAB

BABBAA

BBAAAB

BBAABA

BBABAA

**S5. Pecking Accuracy**

We attempted to estimate the accuracy of the laser grid by simulating pecks of real birds using a stuffed pigeon and a stuffed kea. Using the stuffed birds, we attempted to “peck” as precisely as possible on a 9x9 pixel white stimulus on the screen, with the body position (and thus beak orientation) at different angles. We then analyzed the x/y coordinates recorded by the touch screen. We obtained large differences between extreme pecking values, with highest values equivalent to stimuli composed of 8 tiles. However, these same birds were trained with stimuli up to length 6, and tested with stimuli as small as one tile in width. Therefore, our findings concerning touch screen accuracy reflect our inability to use a stuffed bird as a mean to operate a touch screen, rather than providing information on the accuracy of the birds’ pecking positions. For instance, kea adapted their pecking behaviour by employing the highly flexible upper bill and brushed tongue tip, which was not possible to simulate with a stiff, stuffed bird. Training trials show that birds can be 800% more accurate than our calculations. Assuming a decrease in accuracy, together with concordance between statistical and pecking data by chance, would constitute an extremely unparsimonious explanation.
